# Activated Stat5 trafficking Via Endothelial Cell-derived Extracellular Vesicles Controls IL-3 Pro-angiogenic Paracrine Action

**DOI:** 10.1038/srep25689

**Published:** 2016-05-09

**Authors:** Giusy Lombardo, Patrizia Dentelli, Gabriele Togliatto, Arturo Rosso, Maddalena Gili, Sara Gallo, Maria Chiara Deregibus, Giovanni Camussi, Maria Felice Brizzi

**Affiliations:** 1 Department of Medical Sciences, University of Turin, Italy

## Abstract

Soluble factors and cell-derived extracellular vesicles (EVs) control vascular cell fate during inflammation. The present study investigates the impact of Interleukin 3 (IL-3) on EV release by endothelial cells (ECs), the mechanisms involved in EV release and paracrine actions. We found that IL-3 increases EV release, which is prevented by IL-3Ralpha blockade. EVs released upon IL-3 stimulation were able to induce pro-angiogenic signals as shown by chromatin immunoprecipitation (ChIP) assay performed on the promoter region of cyclin D1 and tridimensional tube-like structure formation. We herein demonstrate that these effects rely on the transfer of miR-126-3p, pre-miR-126 and, more importantly, of activated signal transduction and activator of transcription 5 (pSTAT5) from IL-3-EV cargo into recipient ECs. We show, using the dominant negative form (ΔN)STAT5 and an activated STAT5 (1*6STAT5) constructs, that STAT5 drives IL-3-mediated EV release, miR-126-3p and pSTAT5 content. Finally, using EVs recovered from ΔNSTAT5 expressing ECs, we provide evidence that miR-126-3p and pSTAT5 trafficking is relevant for IL-3-mediated paracrine pro-angiogenic signals. These results indicate that IL-3 regulates EC-EV release, cargo and IL-3 angiogenic paracrine action via STAT5. Moreover, these results provide evidence that EC-derived IL-3-EVs can serve as pro-angiogenic clinical delivery wound healing devices.

Interleukin 3 (IL-3) was originally implicated in the survival, proliferation and differentiation of hematopoietic progenitor/stem cells and in the functional activation of mature cells^1^,^2^. However, the contribution of IL-3 to vascular cell proliferation/activation, first suggested by Orazi *et al.*^3^, has recently been extensively documented^4^,^5^,^6^,^7^,^8^. IL-3 is mainly produced by activated T lymphocytes and mast cells^9^,^10^ in physiological and pathological settings^11^. Besides acting on mature endothelial cells (ECs), IL-3 promotes the expansion and arterial specification of endothelial progenitor cells^5^, while also inducing the proliferation and migration of smooth muscle cells (SMCs)^12^. IL-3’s action, both in hematopoietic and vascular cells, mainly depends upon the activation of STAT5s, whose expression is under the control of one of the most relevant vascular-specific small non-coding RNAs (miR), miR-222^8^. The last decade has seen miRs gaining particular attention in their role as post-transcriptional regulators of genes that are involved in cell differentiation, growth and function^13^. Collectively defined as exRNA, miRs, mRNA and long, non-coding RNA (lncRNA) have recently been detected encapsulated in vesicles in circulation^14^,^15^,^16^. Vesicles have distinct biogenesis or cell origin^17^. The term “exosomes” is usually reserved for vesicles derived from the endosomal membrane compartment by exocytosis, whereas “ectosomes/microvesicles” define vesicles generated by the budding of cell plasma membranes^18^. In fact, the more inclusive term “extracellular vesicles” (EVs) has been suggested, given the overlap in characteristics and biological activities that exists between exosomes and ectosomes/microvesicles^19^. EVs have recently emerged as a well-preserved evolutionary mechanism of cell-to-cell communication^19^.

EVs that are released by a given cell type may function within a defined microenvironment (paracrine action) or may even act at a distance from the cell of origin (endocrine action). EVs contain surface proteins and cytoplasmic molecules from their cell of origin and can therefore impact target cell functional capabilities^20^,^21^. In particular, EV biological activity has been linked to the transfer of receptors, lipids, proteins and exRNA that may change both the phenotype and function of recipient cells^14^,^21^,^22^. While EVs are constitutively released by cells, their release can be enhanced^23^ in response to cell activation or stress. However, the signaling mechanisms that account for EV release have only been partially investigated.

The aim of this present study is to investigate whether the inflammatory cytokine IL-3 exerts angiogenic paracrine effects via EC-EV release. Moreover, the signaling pathway(s) associated with IL-3-induced EV release, cargo and biological effects have also been investigated.

## Results

### IL-3 challenge increases the number of EVs released from ECs and improves their pro-angiogenic capability

**Figure 1:**
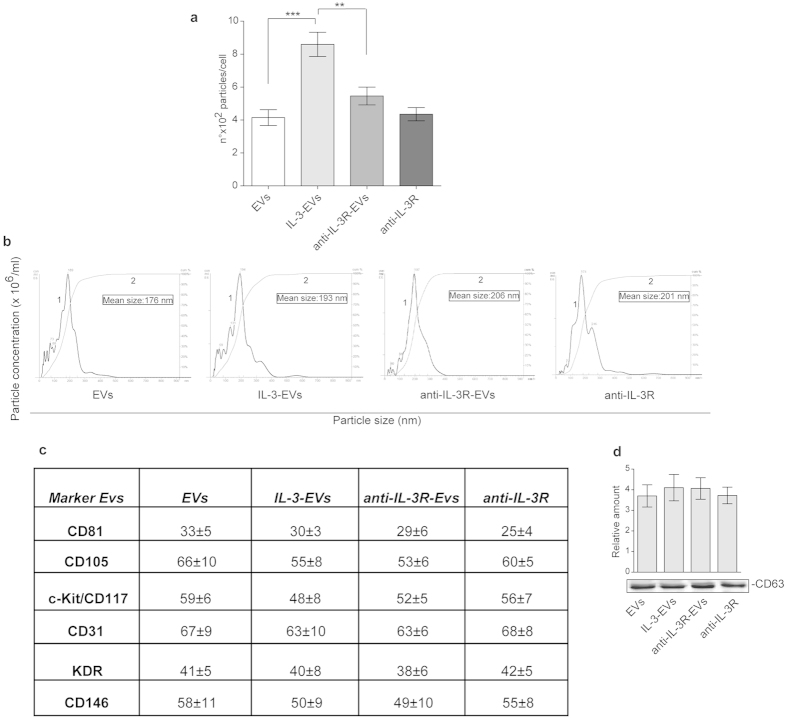
Characterization of EC-derived EVs. (**a**) Number of EV particles (mean ±  SEM) calculated per cell at isolation. Data refer to EVs from ECs (EVs), IL-3-treated ECs (IL-3-EVs) or from ECs pre-treated with the anti-IL3Ralpha blocking antibody (anti-IL-3R) and then stimulated with IL-3 (anti-IL-3R-EVs). The anti-IL-3R alone was also used. The results are representative of four different experiments performed in triplicate (*n* =  4) (***p <  *0.001*, IL-3-EVs vs EVs; **p <  *0.01* and anti-IL-3R-EVs). **(b)** Representative images of NanoSight analyses performed on the 100 k fraction of EC-derived EVs. Curve 1: relationship between particle distribution (left *y* axis) and particle size (*x* axis); curve 2: correlation between cumulative percentage distribution of particles (percentile in right *y* axis) and particle size (*x* axis). The results are representative of four different experiments performed in triplicate (*n* =  4). **(c)** Table resuming FACS analyses of EV surface markers (CD81, CD105, c-Kit/CD117, CD31, KDR and CD146, expressed as percentage of expression ± SD) (*n* =  5). **(d)** CD63 western blotting analysis on EC-derived EVs (*n* =  5).

**Figure 2:**
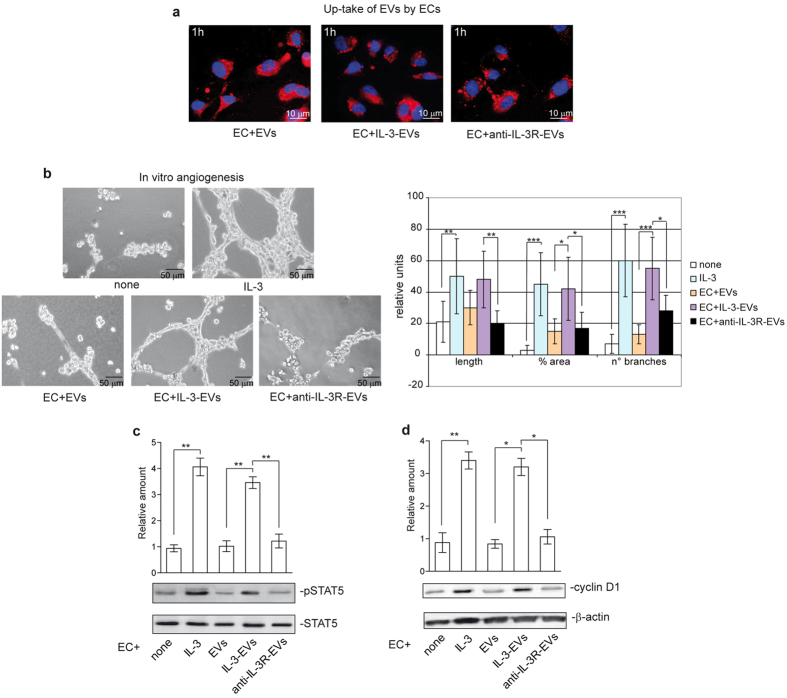
IL-3 improves the pro-angiogenic potential of EC-derived EVs. **(a)** Representative images obtained on a confocal microscope of ECs treated for 1 h with the indicated PKH26-labeled EVs to evaluate up-take of EVs by ECs. The results are representative of four different experiments performed in triplicate (n =  4). Scale bars indicate 10 μ m. **(b)** Representative photomicrographs of an *in vitro* angiogenesis assay, showing tube-like structure formation by ECs, either alone or in the presence of IL-3 (10 ng/ml), EVs, IL-3-EVs or anti-IL-3R-EVs. The quantitative analysis of the number and length of branches and percentage of vessel area (% area) of *in vitro* formed vessel-like structures is reported as mean ±  SD. The results are representative of four different experiments performed in triplicate (n =  4) (for length, **p <  *0.01* IL-3 vs none and IL-3-EVs vs anti-IL-3R-EVs; for % area, ***p <  *0.001* IL-3 vs none, *p <  *0.05* IL-3-EVs vs EVs and anti-IL-3R-EVs; for n° of branches, ***p <  *0.001* IL-3 vs none. IL-3-EVs vs EVs, *p <  *0.05* IL-3-EVs vs anti-IL-3R-EVs). Scale bars indicate 50 μ m. **(c**,**d)** ECs either alone or stimulated with IL-3 (10 ng/ml), EVs, IL-3-EVs or anti-IL-3R-EVs were lysed and analyzed for pSTAT5 (c) and cyclin D1 (d) content. Protein level was normalized to STAT5 and β -actin content, respectively. The results are representative of four different experiments performed in triplicate (*n* =  4) (pSTAT5, **p <  *0.01* IL-3 vs none, IL-3-EVs vs EVs and anti-IL-3R-EVs; cyclin D1, **p <  *0.01* IL-3 vs none, *p <  *0.05* IL-3-EVs vs EVs and anti-IL-3R-EVs).

It has been widely reported that ECs can release EVs^24^,^25^,^26^,^27^. We herein investigate whether differences in EV release can occur when ECs are subjected to inflammatory stimuli, such as IL-3. Figure 1a,b shows that differences in EV number per cell, but not in EV size, were detected by Nanosight. Moreover, EVs evaluated in this study were in the range of nanometers as shown by transmission electron microscopy of EVs (Supplementary Fig. S1a). On these bases, we can reasonably assume that the effect of IL-3 is independent of EC proliferation. The existence of EVs of EC origin was demonstrated by analyzing surface markers (Fig. 1c,d, Supplementary Fig. S2–S4). ECs were pre-treated with an anti-IL-3Ralpha blocking antibody 30 minutes before IL-3 treatment to demonstrate the role of IL-3 in mediating EV release. As reported in Fig. 1a–d, IL-3 blockade inhibits IL-3-induced EV release, without affecting EV size. To evaluate whether EVs released upon IL-3 treatment might act as paracrine factors for neighboring cells, EVs labeled with PKH26 dye were assayed for EC internalization. Figure 2a and Supplementary Fig. S1b show that EVs can be incorporated by ECs. Functional studies were performed using EVs from either unstimulated ECs, from IL-3-treated ECs (IL-3-EVs) or from IL-3-treated ECs that had been pre-incubated with the anti-IL-3Ralpha antibody (anti-IL-3R-EVs). Figure 2b–d shows that IL-3-EVs are more efficient than normal EVs in inducing tube-like structure formation, STAT5 activation and cyclin D1 expression. That this effect depends on IL-3 stimulation is sustained by the inhibitory effect observed when anti-IL-3R-EVs were assayed in the functional assay (Fig. 2b–d). mRNA and protein assays confirmed the absence of IL-3 in IL-3-EVs (Supplementary Fig. S5a,b). The ELISA assay excludes the presence of IL-3 bound to IL-3-EVs (Supplementary Fig. S5c).

### IL-3 treatment leads to EV miR-126-3p accumulation

**Figure 3:**
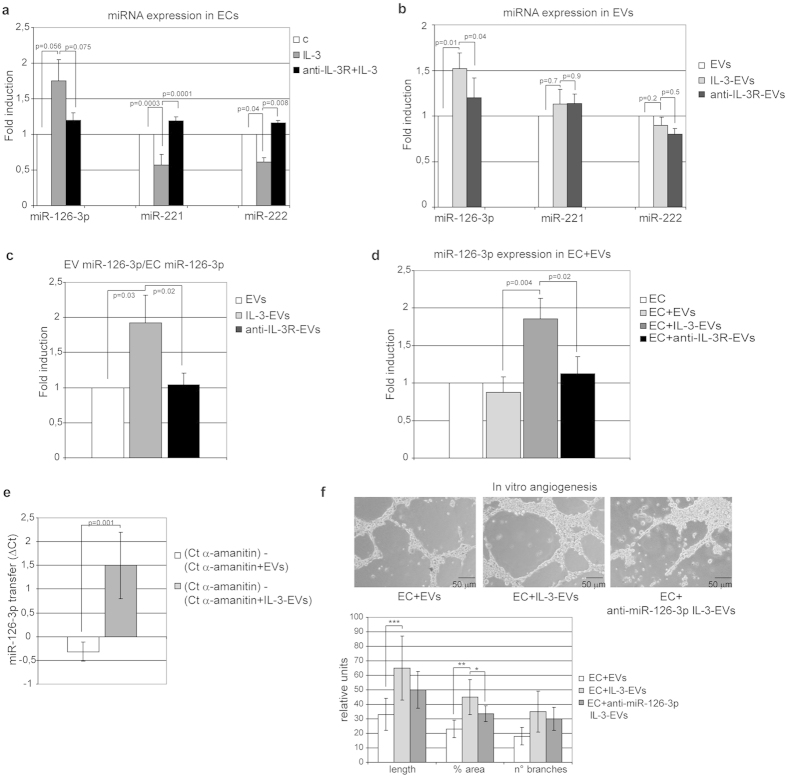
IL-3 dictates the content of miR-126-3p in EC-derived EVs. **(a)** miR‐126-3p, miR-221 and miR-222 expression was evaluated by quantitative real-time PCR (qRT-PCR) on ECs, untreated (**c**) or treated with IL-3 (IL-3) in the presence or in the absence of the anti-IL3Ralpha blocking antibody (anti-IL-3R). Data normalized to RNU6B are representative of four different experiments performed in triplicate (n =  4) (*p* =  *0.056* IL-3 vs none and *p* =  *0.075* IL-3 vs anti-IL3R+ IL-3 for miR-126-3p; *p* =  *0.0003* IL-3 vs none and *p* =  *0.0001* IL-3 vs anti-IL3R+ IL-3 for miR-221: *p* =  *0.04* IL-3 vs none and *p* =  *0.008* IL-3 vs anti-IL3R+ IL-3 for miR-222) **(b)** miR‐126-3p, miR-221 and miR-222 expression was evaluated as above on EVs recovered from ECs, treated as above (*n* =  5) (*p* =  *0.01* IL-3-EVs vs EVs and *p* =  *0.04* IL-3-EVs vs anti-IL3R-EVs for miR-126-3p; *p* =  *0.7* IL-3-EVs vs EVs and *p* =  *0.9* IL-3-EVs vs anti-IL3R-EVs for miR-221: *p* =  *0.2* IL-3-EVs vs EVs and *p* =  *0.5* IL-3-EVs vs anti-IL3R-EVs for miR-222). **(c)** miR‐126-3p expression evaluated on EC-derived EVs was normalized to EC miR‐126-3p content. Data are representative of five experiments performed in triplicate (*n* =  5) (*p* =  *0.03* IL-3-EVs vs EVs and *p* =  *0.02* IL-3-EVs vs anti-IL-3R-EVs). **(d)** miR‐126-3p expression was evaluated by qRT-PCR on ECs untreated or treated with EVs derived from ECs stimulated as above. Data normalized to RNU6B are representative of four different experiments performed in triplicate (*n* =  4) (*p* =  *0.004* EC+ IL-3-EVs vs EC+ EVs and *p* =  *0.02* EC+ IL-3-EVs vs EC+ anti-IL-3R-EVs). **(e)** ECs incubated in the presence of 50 μ g/ml of α -amanitin to inhibit EC transcription were stimulated or not stimulated with EVs or IL-3-EVs. EV-miR-126 transfer was evaluated by q-RT-PCR. The difference in Ct values (Δ Ct) between α -amanitin-treated ECs alone or with the indicated EVs is reported (*p* =  *0.001*) (mean ±  SD). **(f)** Representative photomicrographs of an *in vitro* angiogenesis assay performed on ECs treated as indicated. The quantitative analysis of the number and length of branches and percentage of area of *in vitro* formed vessel-like structures is reported as mean ±  SD. The results are representative of four different experiments performed in triplicate (*n* =  4) (for length, ***p <  *0.001* EC+ IL-3-EVs vs EC+ EVs; for % area **p <  *0.01* ECs+ IL-3-EVs vs ECs+ EVs, *p <  *0.05* ECs+ anti-miR-126 IL-3-EVs vs ECs+ IL-3-EVs). Scale bars indicate 50 μ m.

EV biological activity has been linked to the transfer of proteins, lipids and exRNA, including miRs, into recipient cells^14^,^21^,^22^. In our experimental conditions EVs released by untreated or IL-3-treated ECs showed the presence of small RNA classes, as obtained by a bioanalyzer profile on total RNA of EVs (Supplementary Fig. S1c). We have previously shown that IL-3 changes miR-221/222 content in ECs^8^. We herein evaluate the effect of IL-3 on miR EV cargo. In particular, miR-126-3p and miR-126-5p content^28^,^29^,^30^ was analyzed and compared with its cellular content. miR-221/222 was used as an internal control. miR-221/222, and miR-126-5p were reduced in ECs upon IL-3 stimulation (Fig. 3a and Supplementary Fig. S6a). No significant changes in miR-126-3p cellular content were detected (Fig. 3a). miR content in EVs was therefore evaluated. Figure 3b shows that miR-126-3p, but not miR-221/222, was increased in IL-3-EVs. While reduced EV miR126-5p levels were detected (Supplementary Fig. S6b). As expected, IL-3R blockade prevented the transfer of miR-126-3p into EVs (Fig. 3b) and returned miR126-5p to basal levels (Supplementary Fig. S6b). A significant increase of miR126-3p IL-3-EV content was found even when normalized for its cellular content (Fig. 3c) or when snoRNU48 house-keeping gene was used (data not shown). As only miR126-3p was increased in EVs and its increased expression correlates with angiogenesis^28^, transfer of miR-126-3p into recipient ECs was evaluated to investigate its role in IL-3-EV-mediated biological response. Data reported in Fig. 3d demonstrate that, indeed, miR-126-3p content increased in ECs subjected to IL-3-EVs. It has been reported that EVs from different stem cell sources contain miRs in their precursor form^31^,^32^. We therefore investigated this possibility in our experimental conditions. Indeed, pre-miR-126 was significant increased in IL-3-EVs and its transfer into EVs could be prevented by IL-3R blockade (Supplementary Fig. S6c). That miR-126-3p and pre-miR-126 could be transferred from IL-3-EV cargo into ECs is sustained by the results obtained pre-treating ECs with α –amanitin before IL-3-EV challenge (Fig. 3e and Supplementary Fig. S6d). Functional studies which investigate the possibility that miR-126-3p transfer contributes to the IL-3-EV-mediated pro-angiogenic effect were performed using EVs that had been depleted of miR-126-3p (Supplementary Fig. S7). As shown in Fig. 3f, tube-like structure formation was impaired, but not completely abrogated upon the use of miR-126-3p-depleted IL-3-EVs.

### IL-3 drives pSTAT5 EV content and IL-3-EV biological effects

**Figure 4:**
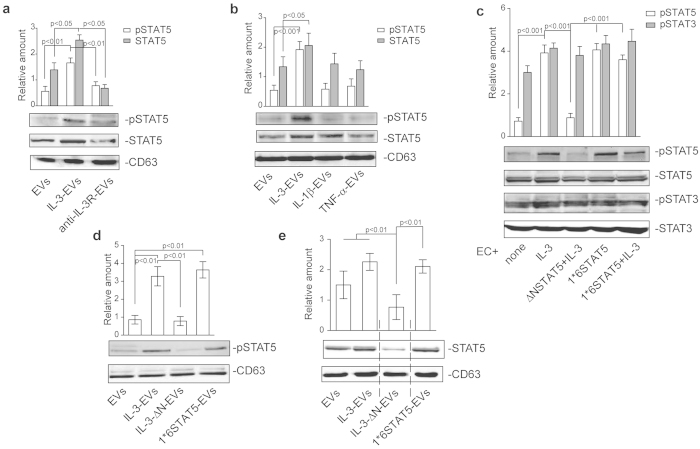
IL-3 treatment drives pSTAT5 EV content. **(a)** EVs recovered from ECs, untreated (EVs) or treated with IL-3 (IL-3-EVs), in the presence or in the absence of anti-IL3Ralpha blocking antibody (anti-IL-3R-EVs) were analyzed by western blotting for pSTAT5 and STAT5 content and normalized to CD63 content. The results are representative of four different experiments performed in triplicate (*n* =  4) (*p* <  *0.01* IL-3-EVs vs EVs and anti-IL-3R-EVs for pSTAT5; *p* <  *0.05* IL-3-EVs vs EVs and anti-IL-3R-EVs for STAT5). (**b**) EVs recovered from untreated or IL-3-, IL-1β - or TNF-α -treated ECs were lysed and analyzed for pSTAT5 and STAT5 content and normalized to CD63 content (*n* =  4) (*p* <  *0.001* IL-3-EVs vs EVs for pSTAT5; *p* <  *0.05* IL-3-EVs vs EVs for STAT5). **(c)** Cell extracts from ECs, transfected or not with Δ NSTAT5 or 1*6STAT5 constructs for 48 h, and treated as indicated, were subjected to SDS-PAGE to evaluate pSTAT5, STAT5, pSTAT3 and STAT3 content. Data are representative of four different experiments performed in triplicate (*n* =  4) (*p* <  *0.001* IL-3 vs none, Δ NSTAT5 vs IL-3, 1*6STAT5 and 1*6STAT5+ IL-3 vs Δ NSTAT5). **(d**,**e)** EVs recovered from untreated, IL-3-treated or Δ NSTAT5-transfected ECs in the presence of IL-3 (IL-3-Δ N-EVs) or 1*6STAT5-transfected ECs (1*6STAT5-EVs) were analyzed for pSTAT5 (d) and STAT5 (e) content and normalized to CD63 (*n* =  4) (*p* <  *0.01* IL-3-EVs vs EVs and IL-3-Δ N-EVs, 1*6STAT5-EVs vs EVs for pSTAT5; *p* <  *0.01* IL-3-Δ N-EVs vs all experimental groups for STAT5).

**Figure 5:**
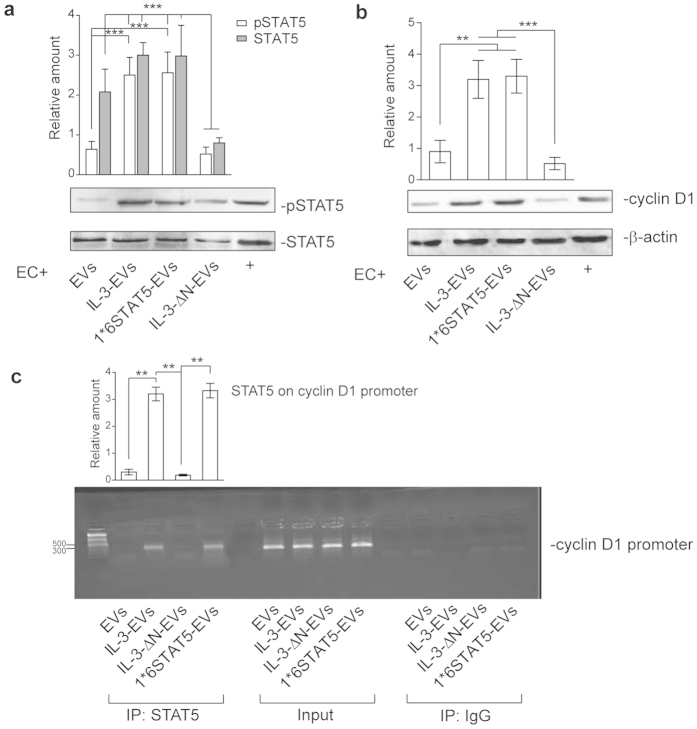
EV pSTAT5 content leads to the formation of a STAT5/cyclin D1 transcriptional complex. **(a**,**b)** Cell extracts from ECs treated with EC-derived EVs, as indicated, were analyzed for pSTAT5 and STAT5 (**a**) or for cyclin D1 (**b**) and β -actin content. The results are representative of four different experiments performed in triplicate (*n* =  4) (***p <  *0.001* EC+ IL-3-EVs and 1*6STAT5-EVs vs EVs, EC+ IL-3-Δ N-EVs vs IL-3-EVs and 1*6STAT5-EVs for pSTAT5 in a; ***p <  *0.001* EC+ IL-3-Δ N-EVs vs all experimental groups for STAT5 in a; **p <  *0.01* EC+ IL-3-EVs and 1*6STAT5-EVs vs EVs, ***p <  *0.001* EC+ IL-3-Δ N-EVs vs IL-3-EVs and 1*6STAT5-EVs for cyclin D1 in b). **(c)** Representative ChIP analysis of STAT5 binding to the cyclin D1 promoter region using ECs treated for 24 h as indicated; data, normalized to the corresponding input values (mean ±  SEM), are reported as relative amount of PCR products (*n* =  4) (**p <  *0.01* EC+ IL-3-EVs vs EVs, IL-3-Δ N-EVs vs EC+ IL-3-EVs and 1*6STAT5-EVs).

**Figure 6:**
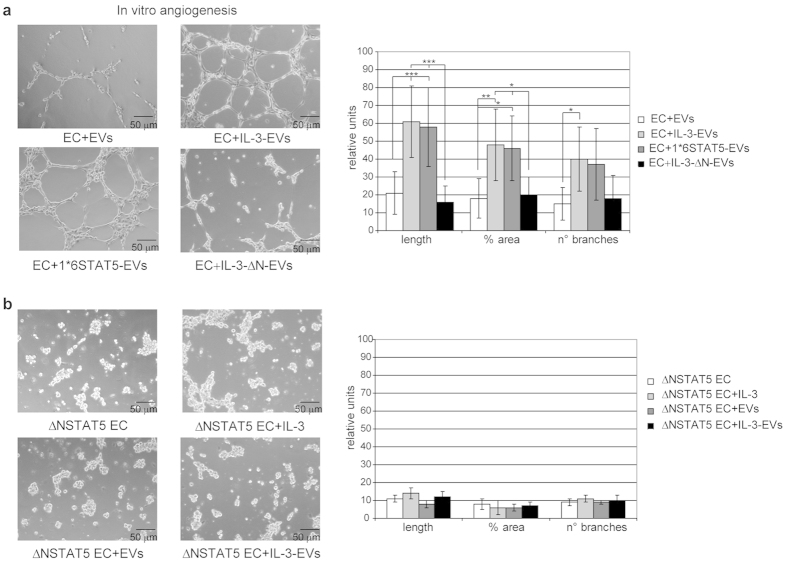
EV pSTAT5 content mediates IL-3-EV biological effects. **(a**,**b)** Representative photomicrographs of an *in vitro* angiogenesis assay performed on ECs treated with EC-derived EVs, as indicated, in a or on ECs transfected with Δ NSTAT5 (Δ N) and treated as indicated in (**b**). Quantitative analysis of *in vitro* formed vessel-like structures (number and length of branches, percentage of area) is reported as mean ±  SD. The results are representative of four different experiments performed in triplicate (*n* =  4) (for length, ***p <  *0.001* EC+ IL-3-EVs and 1*6STAT5-EVs vs EVs, EC+ IL-3-Δ N-EVs vs IL-3-EVs and 1*6STAT5-EVs; for % area **p <  *0.01* EC+ IL-3-EVs vs EVs, *p <  *0.05* ECs+ 1*6STAT5-EVs vs EVs, EC+ IL-3-Δ N-EVs vs IL-3-EVs and 1*6STAT5-EVs; for n° of branches, *p <  *0.05* ECs+ IL-3-EVs vs EVs). Scale bars indicate 50 μ m. (**a**,**b**).

A growing amount of evidence indicates that the transfer of proteins into recipient cells could also account for EV biological response^17^,^18^,^19^,^20^. Thus, we have also evaluated the content of one of the most relevant IL-3 signaling molecules in EVs, STAT5^5^,^8^,^33^. We found slightly increased STAT5 content in IL-3-EVs than in EVs. By contrast, we demonstrate that IL-3 drives pSTAT5EV content, as shown in Fig. 4a. pSTAT5 was not found in EVs that had been recovered from IL-1β – and TNFα -stimulated ECs (Fig. 4b). In order to evaluate the role of IL-3-EV pSTAT5 cargo, biochemical and functional studies were performed using EVs recovered from ECs that were transfected with the Δ NSTAT5 construct and stimulated with IL-3 (IL-3-Δ N-EVs) or that express the constitutive active STAT5A (1*6STAT5-EVs). Transfection efficacy and specificity were confirmed by western blot analysis. STAT5 activation, unlike in STAT3, was regulated by transfection (Fig. 4c). STAT5 content was further evaluated in EVs recovered from transfected ECs. Interestingly, 1*6STAT5-EVs recapitulated IL-3-EV protein cargo (Fig. 4d,e) while the IL-3-Δ N-EVs were impaired in both STAT5 and pSTAT5 content. Further to these results, only ECs stimulated with 1*6STAT5-EV underwent STAT5 activation and cyclin D1 expression (Fig. 5a,b). IL-3 was added to ECs subjected to 1*6STAT5-EV without further effects (data not shown). Thus, we inquired whether IL-3-EV–elicited cyclin D1 expression depended on STAT5-cyclin D1 gene targeting. A ChIP assay, which was performed on the promoter region of cyclin D1^34^, clearly demonstrates that the increased level of activated STAT5 leads to the formation of a STAT5 transcriptional complex which binds to the cyclin D1 promoter (Fig. 5c). Finally, functional experiments performed using either IL-3-Δ N-EVs or 1*6STAT5-EVs demonstrated that IL-3-Δ N-EV-stimulated ECs have an impaired capability to form tube-like structures. Conversely, 1*6STAT5-EVs recapitulated the effect of IL-3-EVs (Fig. 6a). Moreover, the relevance of pSTAT5 intracellular content in regulating EC functional activities was sustained by the observation that both IL-3 and IL-3-EVs failed to promote tube-like structure formation when a Δ NSTAT5 construct was transfected into recipient cells (Fig. 6b). These data as well as the observation that IL-3-EVs did not induce IL-3 expression in ECs (Supplementary Fig. S5a) support the notion that IL-3 transfers a functional transcriptional factor to neighboring ECs, by means of EVs, that could alternatively direct its biological response.

### STAT5 dictates the release of EV and miR-126-3p cargo upon IL-3

**Figure 7:**
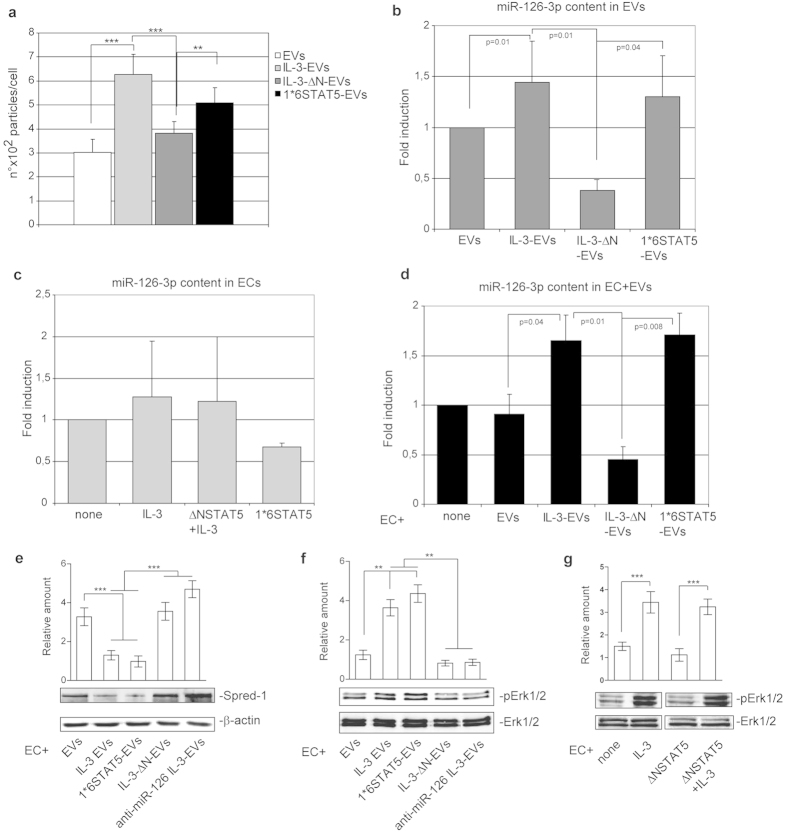
STAT5 dictates the release of EV and miR-126-3p cargo in response to IL-3 stimulation. **(a)** Number of EV particles (mean ±  SEM) calculated per cell at isolation. Data refer to EVs recovered from untreated or IL-3-treated ECs or from ECs transfected with Δ NSTAT5, in the presence of IL-3, or with 1*6STAT5 constructs for 48 h. Results are representative of four different experiments performed in triplicate (*n* =  4) (***p <  *0.001*, IL-3-EVs vs EVs, IL-3-Δ N-EVs vs IL-3-EVs; **p <  *0.01* 1*6STAT5-EVs vs IL-3-Δ N-EVs). **(b)** miR‐126-3p, normalized to RNU6B, was evaluated on EVs recovered from ECs, treated as above (*n* =  4) (*p* =  *0.01* IL-3-EVs vs EVs, *p* =  *0.01* IL-3-Δ N-EVs vs IL-3-EVs, *p* =  *0.04* 1*6STAT5-EVs vs IL-3-Δ N-EVs). **(c)** miR‐126-3p was evaluated by qRT-PCR on ECs untreated or treated with IL-3 or transfected with Δ NSTAT5, in the presence of IL-3, or with 1*6STAT5 constructs. Data normalized to RNU6B are representative of four different experiments performed in triplicate (*n* =  4). **(d)** miR‐126-3p expression was evaluated by qRT-PCR on ECs untreated or treated with EVs derived from ECs as indicated. Data normalized to RNU6B are representative of four different experiments performed in triplicate (*n* =  4) (*p* =  *0.04* EC+ IL-3-EVs vs EC+ EVs, *p* =  *0.01* EC+ IL-3-Δ N-EVs vs IL-3-EVs, *p* =  *0.008* ECs+ 1*6STAT5-EVs vs IL-3-Δ N-EVs). **(e**,**f)** Cell lysates from ECs treated with EC-derived EVs, as indicated, were analyzed for Spred-1 (**e**) or for pErk1/2 (**f** ) content and normalized to β -actin or Erk1/2 content, respectively (*n* =  4) (***p <  *0.001* EC+ IL-3-EVs and 1*6STAT5-EVs vs EVs, EC+ IL-3-Δ N-EVs and anti-miR-126 IL-3-EVs vs IL-3-EVs and 1*6STAT5-EVs for Spred-1 in e; **p <  *0.01* EC+ IL-3-EVs and 1*6STAT5-EVs vs EVs, EC+ IL-3-Δ N-EVs and anti-miR-126 IL-3-EVs vs IL-3-EVs and 1*6STAT5-EVs for pErk1/2 in (**f** ). **(g)** Cell extracts from ECs untreated or treated with IL-3 for 15 minutes, or from Δ NSTAT5-transfected ECs, in the presence or in the absence of IL-3, were analyzed for pErk1/2 content, normalized to Erk1/2 content (*n* =  4) (***p <  *0.001* EC+ IL-3 vs none, EC+ Δ NSTAT5+ IL-3 vs NSTAT5).

The results reported above led us to investigate whether STAT5 is able to control IL-3-mediated EV release and miR-126-3p content. To this end, the number of EVs released from transfected ECs was analyzed. Figure 7a shows that IL-3-Δ NSTAT5 construct expression significantly reduces the number, but not the size (Supplementary Fig. S8a) of EVs released in response to IL-3. That EC-EV release depends on IL-3-mediated STAT5 activation was further sustained by the number of EVs recovered from IL-3-stimulated ECs pre-treated with a specific JAK2 inhibitor (Supplementary Fig. S8a–c). EV miR-126-3p cargo was also analyzed. Data reported in Fig. 7b demonstrate that IL-3-Δ N-EVs carry reduced miR-126-3p content. No significant differences in miR-126-3p cellular content were found after transfection (Fig. 7c). Moreover, the transfer of pSTAT5 (Fig. 5a) and miR-126-3p into ECs that had been stimulated with IL-3-Δ N-EVs was reduced (Fig. 7d). As expected, the number of EVs released by ECs expressing the 1*6STAT5 construct was almost comparable to values obtained upon IL-3 treatment. Likewise, miR-126-3p EV cargo and miR-126-3p transfer into ECs were rescued even in the absence of IL-3 stimulation (Fig. 7b,d). These results demonstrate that the activation of the STAT5 signaling pathway is crucial for IL-3-mediated EV release and cargo.

### IL-3-EV miR-126-3p content is relevant for Erk1/2 activation

The reduced IL-3-Δ N-EV miR-126-3p content led us to evaluate the contribution of miR-126-3p to IL-3-EV angiogenic property. The miR-126-3p target, Spred-1^28^,^35^, was therefore evaluated in ECs treated with IL-3-Δ N-EVs and compared with those stimulated with miR-126-3p -depleted IL-3-EVs. Results reported in Fig. 7e demonstrate that Spred-1 increased under both experimental conditions, while it was almost undetectable in ECs stimulated with IL-3-EVs or 1*6STAT5-EVs. The activation of Erk1/2 inversely correlates with Spred-1 expression (Fig. 7f). These data provide further evidence that EV miR-126-3p and pSTAT5 content cooperate in mediating IL-3-EV paracrine effects. An independent cooperation between these signaling pathways is also supported by the observation that IL-3 was still able to induce Erk1/2 activation in ECs depleted of STAT5 (Fig. 7g).

## Discussion

We have shown in the present study that IL-3 promotes pro-angiogenic paracrine effects via EC-derived EVs. In particular, we have noticed that IL-3 upholds EC-EV release and increases EV miR-126-3p and pSTAT5 content. This translates into the transfer of functional miR-126-3p and STAT5 into ECs which leads to cyclin D1 transcription and tridimensional tube-like structure formation. We also found that functional STAT5 drives EV release, IL-3-EV cargo and biological activity. Finally, we noticed that enrichment in functional STAT5 and miR-126-3p is sufficient to facilitate the signaling functions of IL-3-EVs. These results indicate that IL-3 impacts on EC-derived EV functional capability and provide evidence for IL-3-EVs’ involvement, as paracrine factors, in the physiological and pathological processes of wound healing.

IL-3, originally described as a hematopoietic growth factor, also exerts relevant action on the vascular wall during inflammation^36^. Its role in inflammation has been underlined further by Weber *et al.*^37^. IL-3, mainly produced by activated lymphocytes, can induce EC activation^38^, smooth muscle cell proliferation^12^, endothelial progenitor cell expansion^5^ as well as neoangiogenesis^8^. In the present study, we investigated IL-3-mediated paracrine actions. There is increasing evidence to suggest that EVs act as a well-preserved cell-to-cell communication mechanism which impacts on both neighboring and remote cells in physiological and pathological settings^19^,^21^. EVs have different effects on the endothelium depending on their cell of origin; however no data on EC-derived EV paracrine effects in response to IL-3 are currently available. We have shown herein that the number of EC-derived EVs can be increased by stimulating ECs with IL-3 and that IL-3-EVs, independently of IL-3 (IL-3 was detected neither in IL-3-EVs nor in ECs upon IL-3-EV treatment), are effective in inducing pro-angiogenic signals when used to stimulate ECs. As a matter of fact, IL-3-EVs induce the activation of STAT5 and the expression of cyclin D1 as well as tridimentional, tube-like structure formation. We also demonstrate that IL-3-EV actions rely on the transfer of their cargo into ECs. Increased miR-126-3p content can be detected in ECs stimulated with IL-3-EVs. Selective EV-miR and pre-miR availability has been suggested to represent a rapid means of regulating gene expression^14^,^15^,^16^,^31^,^32^. As a matter of fact, our functional studies and the activation of the Erk1/2 signaling pathway indicate that functional miR-126-3p and its precursor form, released into the tissue microenvironment via EVs, in response to IL-3, could be transferred into recipient cells and rapidly induce pro-angiogenic signals.

We did not investigate the specific role of miR126-5p in our experimental conditions. However, the finding that miR126-5p is down-regulated in response to IL-3 is in agreement with the observation that low EC miR-126-5p levels could participate in the control of leucocyte trafficking^30^ and with our original data^38^ demonstrating that IL-3 can promote EC activation and leukocyte adhesion. Our data suggest that also EVs, via paracrine mechanisms, could participate to such effect. However, further studies are required to address this issue.

EVs can possess pro-angiogenic properties^26^,^39^ when transferring metalloproteinases MMP-2 and MMP-9, which support matrix degradation and new blood vessel formation^35^,^40^. In addition, their protein content means that EVs have been also considered signalosomes^41^,^42^. Indeed, EVs may trigger intracellular signaling pathways by engaging receptors, ligands and by transferring functionally active receptors, such as CCR5^43^, EGFRvIII^44^ and MET^45^. We herein demonstrate that, upon IL-3 stimulation, EVs released from ECs carry the IL-3 specific signaling molecule STAT5, which triggers a specific signaling pathway when transferred into recipient cells. Data on death receptor ligands^46^,^47^, have suggested that functional molecules are more active when delivered by EVs than in their soluble forms. Indeed, our data demonstrate that IL-3-EVs mainly transfer the activated form of STAT5 to recipient cells, unlike EVs released by other inflammatory stimuli such as IL-1β and TNFα . Although we have no additional evidences on EC-derived EV content upon IL-1β – and TNFα –treatment, we can speculate that IL-3-EV cargo might merely reproduce the intracellular pSTAT5 content. Alternatively, IL-3-EV cargo might reflect the relevance of pSTAT5 for IL-3-mediated paracrine actions. As a matter of fact, STAT5 has been reported to strictly control the proliferation and arterial specification of endothelial progenitor cells, thus resulting in neovessel formation^5^. This effect is mediated by cyclin D1 expression^48^. Similarly, IL-3-EVs are able to induce the expression of cyclin D1 by transferring the activated STAT5 to neighboring ECs. The concept that the transfer of activated STAT5 is crucial to cyclin D1 transcription in response to IL-3-EVs is sustained by the following observations: i. IL-3-Δ N-EVs, depleted of pSTAT5, failed to induce cyclin D1 transcription; ii. 1*6STAT5-EVs, containing high levels of activated STAT5, were able to induce cyclin D1 transcription even in the absence of IL-3. Proof of the role of the activated STAT5 transferred by IL-3-EVs in promoting pro-angiogenic signals has also been provided by functional studies performed with IL-3-Δ N-EVs and 1*6STAT5-EVs. Tridimentional tube-like structure formation, in response to IL-3-EVs, can be recapitulated by 1*6STAT5-EVs even in the absence of IL-3. Finally, the failure of both IL-3 and IL-3-EVs to promote pro-angiogenic signals in recipient ECs depleted of STAT5 provides the proof of concept that pSTAT5 intracellular content^5^,^34^ is crucial for EC functional activities.

EC activation most likely contributes to changing EV levels and contents, ultimately influencing their functions. It has been reported that EV release is enhanced in ECs and leukocytes exposed to inflammatory stimuli^49^,^50^, however the molecular mechanisms regulating these processes have so far remained elusive. In the present study, we demonstrate that STAT5 activation by IL-3 also controls the release and trafficking of EVs from parental to recipient cells. More importantly, we demonstrate that STAT5 activation, in an inflammatory microenvironment containing IL-3, provides the machinery for ECs to arrange their EV miR-126-3p cargo without disturbing miR-126-3p intracellular content. This implies that IL-3, after binding to its receptor, can both directly influence cell behavior as well as driving cell-to-cell signaling through EV secretion and reuptake by neighboring cells. Moreover, the failure to obtain further biological effects from using IL-3-EVs together with IL-3 (data not shown) sustains the notion that IL-3-EVs can transfer the relevant machinery to boost pro-angiogenic signals in the tissue site of inflammation by themselves.

**Figure 8:**
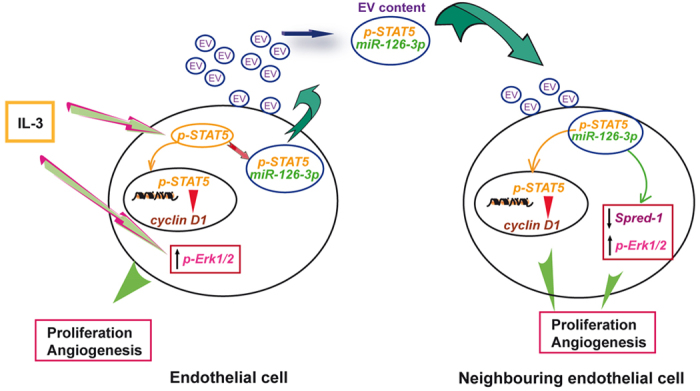
Schematic representation of IL-3 paracrine actions via EC-EVs. IL-3 stimulation leads to Erk1/2 activation. IL-3 stimulation leads to STAT5 activation and cyclin D1 transcription^48^. On the other hand, IL-3-mediated STAT5 activation is involved in the control of EV cargo (at least of pSTAT5, miR-126-3p EV content) and EV release (left panel). The released vesicles are internalized by neighboring ECs. The transfer of EV-miR126-3p and pSTAT5 into recipient ECs independently promotes pro-angiogenic signals (right panel).

In physiological conditions, quiescent endothelium secretes EVs that inhibit monocyte activation and suppress EC activation^51^. In the present study, we provide evidence to show that EC-EVs, in an inflammatory setting containing IL-3, mediate the horizontal transfer of bioactive proteins, miRs and their precursors, such as pSTAT5, miR-126-3p and pre-miR-126, which independently, could boost the local adaptation to wound healing (Fig. 8). Moreover, since EVs have gained significant interest as potential biomarkers and pharmacological targets of vascular damage, our data suggest that pSTAT5 may represent a specific IL-3-EV protein signature.

## Methods

[Table t1]

### Cell cultures

Endothelial cells (ECs) were isolated from the human umbilical vein by Trypsin treatment within 4 hours of delivery (0.1%, w/v), were cultured in M199 with the addition of 20% (v/v) BCS and 5 ng/ml of bFGF and used at early passage (II-III)^52^. To collect EVs, ECs were starved in M199 deprived of BCS for 24 h, treated or not with IL-3 (10 ng/ml). Cell viability was evaluated by trypan blue at the end of each experiment (94 ±  5% viable cells/experiment). Where indicated, ECs were pre-incubated with the anti-IL-3R antibody (20 μ g/ml) 30 minutes before IL-3 treatment. In selected experiments, EVs were recovered from oligonucleotide- or construct-transfected ECs, as described below, or from IL-1β (10 ng/ml) or TNF-α (10 ng/ml) treated ECs. In addition, EVs were recovered from IL-3-stimulated ECs pretreated with JAK2 inhibitor (20μ M). ECs were also cultured in M199 without BCS for 24 h in the presence of EC-derived EVs, as indicated. In preliminary studies a dose response curve was performed to evaluate the number of EVs needed to obtain the best biological response. We tested different EV numbers (from 1 to 8 ×  10^3^) per target cell (10 ×  10^4^ cells/well/2 ml). We found that 5 ×  10^3^ EVs/target cell was the most effective EV dose. Thus, 5 ×  10^3^ EVs/target cell were used throughout the study. All experiments were performed in accordance with the European Guidelines and policies and approved by the Ethical Committee of the University of Turin. Ethical approval was also obtained from Azienda Ospedaliero-Universitaria (AOU), Città della Salute e della Scienza di Torino, Italy. Informed consent was obtained from all subjects in accordance with the Declaration of Helsinki.

### Isolation of EC-derived EVs

ECs were cultured in M199, without BCS, for 24 h in order to collect EVs from supernatants. After being centrifuged at 3 k g for 30 minutes to remove debris, cell-free supernatants were submitted to differential ultracentrifugation at 10 k and 100 k g (Beckman Coulter Optima L-90K ultracentrifuge; Beckman Coulter, Fullerton, CA) for 3 h at 4 °C. EVs were either used fresh or were stored at − 80 °C after re-suspension in M199 which was supplied with 1% DMSO^53^. Frozen EVs were washed and pelleted by 100 kg ultracentrifugation to remove DMSO before cellular experiments. No difference in biological activity was observed between fresh and stored EVs. The protein content of EVs was quantified using the Bradford method (Bio-Rad, Hercules, CA, USA). Any possible contamination was tested using a Limulus amebocyte assay (concentration < 0.1 ng/ml) (Charles River Laboratories, Inc., Wilmington, MA, USA). EV size distribution analysis was performed using a NanoSight LM10 (NanoSight Ltd, Minton Park UK). The particles in the samples were illuminated using a laser light source and the scattered light was captured by camera and analyzed using Nanoparticle Tracking Analysis (NTA). NTA automatically tracked and sized particles according to Brownian motion and the diffusion coefficient (Dt). Results were displayed as a frequency size distribution graph and outputted to a spreadsheet.

### Characterization of EC-derived EVs

Fluorescence-activated cell sorting (FACS) analysis of EC-derived EVs was performed as indicated^54^ using antibodies direct to CD81, CD31, CD105, CD146, KDR and c-Kit (also known as CD117), directly or indirectly conjugated with FITC or-PE fluorocrome. FITC or PE mouse non-immune isotypic IgG (Dako Cytomation) was used as a control. Briefly, the FACS analysis of EC-derived EVs was performed using a Guava easyCyteTM Flow Cytometer (Millipore, Germany). FITC-or PE-conjugated antibodies were added to a suspension of EVs (250 particles/ml in 100μ l) for 15 min at 4 °C in order to perform flow cytometric analysis by FACS of EVs. The volume was increased up to 500μ l with a FACS flow and the expression of surface markers was evaluated. Surface marker expression is reported as percentage of expression ± SD and representative histogram and dot plot analysis are reported in the Supplementary Figures. CD63 content in EVs was analyzed by western blot.

### EV internalization by ECs

The internalization of EVs into ECs was evaluated using confocal microscopy (LSM5-PASCAL; Zeiss, Oberkochen, Germany). A pool of approximately 2.5 ×  10^8^ EV particles was labeled with red fluorescent PKH26 dye (2 μ l/ml) for 30 min at 37 °C and then EVs were washed and ultracentrifuged at 100 k g for 1 h at 4 °C. EV pellets were suspended in M199 and added to ECs (1 ×  10^6^) for several hours in order to detect their internalization (photomicrographs reported in the results show EV internalization after 1 h)^55^. Z*-*stack confocal microscopy EC images were also obtained.

### Transmission electron microscopy

Transmission electron microscopy (TEM) was performed on EVs isolated by ultracentrifugation resuspended in PBS, placed on 200 mesh nickel formvar carbon coated grids (Electron Microscopy Science, Hatfield, PA) and left to adhere for 20 min. Grids were then incubated with 2.5% glutaraldehyde containing 2% sucrose and after washings in distilled water the EVs were negatively stained with NanoVan (Nanoprobes, Yaphank, NK, USA) and observed by Jeol JEM 1010 electron microscope (Jeol, Tokyo, Japan).

### Western blot analysis

ECs and EVs were lysed and protein concentrations obtained as previously described^56^. 50 μ g protein for cells and 10 μ g for EVs were subjected to SDS-PAGE, transferred into nitrocellulose membranes and processed as previously described^56^. Densitometric analysis was used to calculate the differences in the fold induction of protein levels which were normalized to STAT5, STAT3, β -actin, CD63 or Erk1/2 content. Values are reported as relative amounts.

### RNA isolation and quantitative real-time PCR (qRT-PCR)

Total RNA was isolated from ECs using the TRIzol reagent (Invitrogen) and from different EVs using the mirVana RNA isolation kit (Ambion), according to manufacturer’s instructions. RNA was quantified spectrophotometrically (Nanodrop ND-1000, Wilmington, DE, USA) because intact 18S and 28S rRNAs were difficult to detect in the EVs. RNA quality was assessed on an Agilent 2100 Bioanalyzer (Agilent Technologies Inc., Santa Cruz, CA) using tan Agilent small RNA kit (Agilent Technologies). RNA from cells and EVs was then reverse-transcribed using a TaqMan microRNA RT kit, specific for miR-126-3p, miR-221 and miR-222, and subjected to quantitative real-time-PCR (qRT-PCR) using a TaqMan microRNA assay kit and the ABI PRISM 7700 sequence detection system (Applied Biosystems, Foster City, CA, USA). miR expression was normalized to the small nuclear RNA, RNU6B or RNU48. In selected experiments, loss-of-function approaches were performed in ECs that had been transfected for 48 h with the anti-miR negative control or the anti-miR-126-3p antagomir (Applied Biosystems). EV isolation was then obtained from ECs that had been depleted of miR-126-3p. In selected experiments, total RNA, isolated from ECs by TRIzol or from EVs by mirVana RNA extraction kit after DNAse digestion, was subjected to reverse RT-PCR with specific primers: IL-3 (sense, 5′ -GCCCGTCCTGCTCCTGCTCCA-3′ ; antisense, 5′ -CCGGAATTCA-TTC-CAGTCAC-3′ ) and β -actin (sense, 5′ -GGTCATCTTCTCGCGGTTGGCCTTGGGGT-3′ ; antisense, 5′ -CCCCAGGCACCAGGGCGTGAT-3′ ). Total RNA from IL-3 producing gibbon MLA cell line was used as a control (+ ). In selected experiments, total RNA, isolated from ECs by TRIzol or from EVs by mirVana RNA extraction kit after DNAse digestion, was assessed by qRT-PCR using miScript Reverse Transcription Kit and miScript SYBR Green PCR Kit (both from Qiagen). qRT-PCR was performed using a 96-well StepOne™ Real-Time System (Applied Biosystems). The following specific hsa-miR-126-5p (5′ CATTATTACTTTTGGTAC 3′ ) and hsa-pre-miR-126 (5′ TGGCGACGGGACATTATT 3′ ) primers were used. miR expression was normalized to snoRNA RNU48 (5′ AACTCTGAGTGTGTCGCTGATG 3′ ) and RNU6B (5′ CGCAAGGATGACACGCAA 3′ ).

### Transfer of miR-126-3p and pre-miR-126 from EVs to ECs

In order to analyze miR-126-3p and pre-miR-126 transfer from IL-3-EVs to ECs, miR transfer experiments were conducted as previously described by Yuan^57^. Approximately 5 ×  10^5^ cells/well of ECs were incubated for 24 h with EVs or IL-3-EVs and a transcription inhibitor, α -amanitin (Sigma, 50 μ g/ml) or with α -amanitin alone^58^ to inhibit transcriptional activation induced by EVs. Total RNA from ECs, treated as above, was isolated and subjected to qRT-PCR for miR-126-3p and pre-miR-126 expression. As an indirect measure of miR transfer, we determined the difference in Ct values (Δ Ct) between α -amanitin treated cells in the absence or in the presence of EVs or IL-3-EVs; a positive value indicated transfer of miR into target cells (mean ±  SD). If no signal was detected, a Ct value of 40 was assigned to the sample.

### Transfection of dominant negative (ΔN) STAT5 construct and activated form of STAT5

In selected experiments, EC cells were transiently transfected with the Δ NSTAT5 construct^5^ or with the activated form of STAT5 (1*6 STAT5A)^59^ and either cultured in the presence or absence of IL-3 (10 ng/ml). The supernatant was collected 48 hours later to isolate EVs and then cells were processed to obtain cell extracts for Western blot analysis or total RNA isolation to evaluate miR-126-3p expression.

### Tube-like structure formation (*in vitro* angiogenesis assay)

24-well-plates were coated with growth factor-reduced Matrigel matrix to analyze tube-like structure formation by ECs, as previously described^35^. Briefly, ECs were seeded on a Matrigel matrix with or without: IL-3 (10 ng/ml), EVs isolated from unstimulated ECs (EVs), IL-3-treated ECs (IL-3-EVs), IL-3+ anti-IL-3R antibody-treated ECs (anti-IL-3R-EVs), IL-3+ Δ NSTAT5-treated ECs (IL-3-Δ N-EVs) and 1*6STAT5-treated ECs (1*6STAT5-EVs). In selected experiments the angiogenesis assay was performed in ECs transfected with the Δ NSTAT5 construct and stimulated with IL-3, EVs, or IL-3-EVs. After 6 h, the formation of tube-like structures was evaluated. Phase-contrast images (20X magnification) were recorded and the total length of network structures, the number of branches and the percentage of vessel area over total area per field were measured using a MicroImage analysis system (Casti Imaging, Italy) in five random fields and expressed as a ratio to the respective control (mean ±  SD).

### Chromatin Immunoprecipitation Assay

The chromatin Immunoprecipitation (ChIP) assay was performed on ECs treated with various EVs as indicated in the Results section, using Magna ChIP A kit (Millipore)^34^, according to the vendor’s instructions. Briefly, ECs, treated as indicated, were cross-linked with 1% formaldehyde and quenched before harvest and sonication. The sheared chromatin was immunoprecipitated with the anti-STAT5 antibody or control IgG on protein G Sepharose magnetic beads. The eluted IP were digested with proteinase K, and DNA was extracted which underwent PCR with primers specific for cyclin D1 promoter region: sense, 5′ - GATGCAGTCGCTGAGATTCTT-3′ ; antisense, 5′ -TTGCCCCTGTAGTCCGGTTTT-3′ . The non-immunoprecipitated genomic DNA was also analyzed using semi-quantitative real-time PCR and expressed as % of the input.

### Elisa Assay

To evaluate the levels of bound IL-3 on EVs, IL-3-EVs and anti-IL-3R EVs were measured using a colorimetric sandwich enzyme immunoassay (ELISA kit, R&D Systems) as described by the manufacturer’s instructions.

### Statistical analysis

All data are presented as mean ±  SEM, unless otherwise reported. The D’Agostino–Pearson test was used to test normality. Data on the *in vitro* angiogenesis, ELISA and ChIP assays, on RT-PCR, miR expression, loss-of-function experiments, α -amanitin experiments, number of recovered EVs per cell and lastly on the densitometric analysis for Western blots were analyzed using the Student *t* tests for 2-group comparison and using 1-way ANOVA, followed by Tukey’s multiple comparison test, for ≥ 3 groups. The minimum sample size was four experiments performed in triplicate, thus ensuring 90% statistical power between experimental groups, with a probability level of 0.05, two-tailed hypothesis. The cut-off for statistical significance was set at *P* <  0.05 (*P <  0.05, **P <  0.01, ***P <  0.001). All statistical analyses were carried out using GraphPad Prism version 5.04 (Graph Pad Software, Inc).

### Sources of funding

This work has been supported by grants obtained by GC and MFB from the Associazione Italiana per la Ricerca sul Cancro (AIRC) projects IG 12890 and IG 17630 and by grants obtained by MFB from Ministero dell’Istruzione, Università e Ricerca (MIUR) ex 60%.

## Additional Information

**How to cite this article**: Lombardo, G. *et al.* Activated Stat5 trafficking Via Endothelial Cell-derived Extracellular Vesicles Controls IL-3 Pro-angiogenic Paracrine Action. *Sci. Rep.* **6**, 25689; doi: 10.1038/srep25689 (2016).

## Supplementary Material

Supplementary Information

## Figures and Tables

**Table 1 t1:** Reagents and Antibodies.

Description	Purchased from
**Reagents**	
Bovine calf serum (BSA) (A8806), FCS (12138C), FBS (F6178), SDS (L3771), PIPES (P9291), Triton X-100 (T8787), Nonidet P-40 (74385), NaCl (S3014), NaF (S7920), Na_3_OV_4_ (S6508), Na_4_P_2_O_7_ (P8010), MgCl_2_ (M8266), KCl (P9541), HCl (258148), Na-azide (S2002), Hepes (H3375), Tris (T1503), EDTA (E6758), EGTA (E4378), ethanol (51976), aprotinin (A6279), pepstatin A (P5318), PMSF (P7626), DMSO (D8418), PKH26 dye (MINI26-1KT), Medium 199 (M4530), leupeptin (L2884), penicillin-streptomycin (P4333), Trypsin (T4799), α -amanitin (A2263)	Sigma-Aldrich (St Louis, MO, USA)
Protein molecular weight markers (161-0374), Acrylammide (161-0156), polyvinylidene difluoride (PVDF) membranes (162-0115), Bradford reagent (500–0205), ECL (170–5061)	Bio-Rad (Hercules, CA, USA)
Matrigel Basement Membrane Matrix Growth Factor Reduced (356231)	BD Bioscience Pharmingen (Franklin Lakes, NJ, USA)
Lipofectin^®^ Reagent (18292-037), TRIZOL (15596018), hsa-miR-126-3p (002228), RNU6B (001093), hsa-miR-221 (000524), hsa-miR-222 (002276), has-miR-126-3p Anti-miR™ miRNA Inhibitor (AM12841, MIMAT0000445), RNU48 (001006)	Invitrogen^TM^ (Life Technologies Carlsbad, CA, USA; Paisley, UK).
Human recombinant IL-3 (200-03)	PeproTech EC Ltd (London, UK)
Human recombinant IL-1β (200-1B)	
Human recombinant TNF-α (300-01A)	
Human recombinant bFGF (100-18B)	
**Antibodies**	
anti-CD105-FITC (1F-298-T100) anti-CD81-PE (1P-588-T100)	EXBIO Praha AS (Vestec, CR)
anti-CD31-FITC (130-092-654)	Miltenyi Biotec GmbH (Bergisch Gladbach, Germany)
anti-CD146-FITC (5050-F100T)	BioCytex SARL (Marseille France)
anti-Spred-1 (sc-393198)	S. Cruz Biotechnology (Heidelberg, Germany)
anti-pErk1/2 MAPK (sc-16982)	
anti-Erk1/2 MAPK (sc-93)	
anti-β actin (sc-47778)	
anti CD63 (sc-15363)	
anti cyclin D1 (sc-20044)	
anti-goat IgG-FITC (sc-2356)	
anti-rabbit IgG-PE(sc-3739)	
anti-goat IgG-PE (sc-3743)	
anti-KDR (sc-504)	
anti-c-Kit/CD117 (sc-13508)	
anti-STAT5 (sc-835)	
anti-STAT3 (sc-483)	
anti-pSTAT3 (9131S)	Cell Signaling Technology (Danvers MA, USA)
anti-pSTAT5 (9314S)	
Human IL-3 R alpha Antibody (MAB301)	R&D System Inc. (Minneapolis, MN, USA)
anti-rabbit IgG, HRP linked (4050-05)	Southern Biotech (Birmingham, Alabama USA)
anti-mouse IgG, HRP linked (1031-05)	
Tyrphostin AG-490 (19-146)	Merck S.p.a.(Vimodrone, MI, Italy)
anti-mouse IgG-PE (550589)	BD Bioscience Pharmingen (Franklin Lakes, NJ, USA)
anti-mouse and rabbit IgG-FITC (555988 and 554020)	
