# Supplementary material for: Activated Stat5 trafficking Via Endothelial Cell-derived Extracellular Vesicles Controls IL-3 Pro-angiogenic Paracrine Action
Source: Sci Rep. 2016 May 9;6:25689. doi: 10.1038/srep25689 (PMC4860593; doi:10.1038/srep25689)
Supplement: Supplementary Information [file srep25689-s1.doc]

**ACTIVATED STAT5 TRAFFICKING VIA ENDOTHELIAL CELL-DERIVED EXTRACELLULAR VESICLES CONTROLS IL-3 PRO-ANGIOGENIC PARACRINE ACTION.**

Giusy Lombardo, Patrizia Dentelli, Gabriele Togliatto, Arturo Rosso, Maddalena Gili, Sara Gallo, Maria Chiara Deregibus, Giovanni Camussi and Maria Felice Brizzi

**Supplementary Information**

**
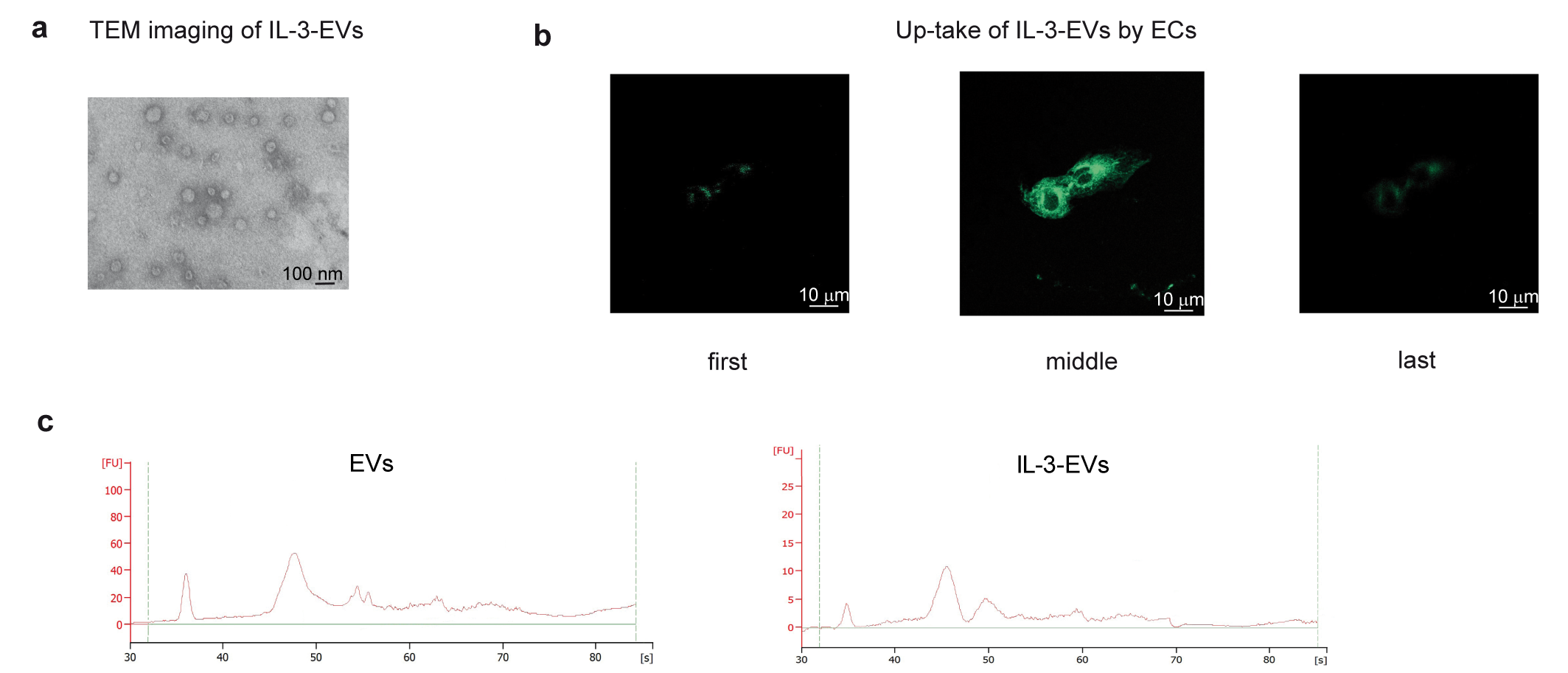
**

**Supplementary Figure S1. (a)** Representative transmission electron microscopy (TEM) imaging of EVs negatively stained with NanoVan. EVs were viewed by JEOL Jem 1010 electron microscope (black line= 100 nm). **(b)**To evaluate EVs up-take by ECs, ECs were treated for 1h with PKH26-labeled IL-3-EVs and analyzed. Representative sections (first-middle-last) of images (Z-stack) obtained on a confocal microscope are reported. Four different experiments performed in triplicate (n=4). Scale bars indicate 10 μm. **(c)** Representative bioanalyzer profile of small RNAs performed on EVs or IL-3-EVs, showing an enrichment of small RNAs of the miRNA size.

**
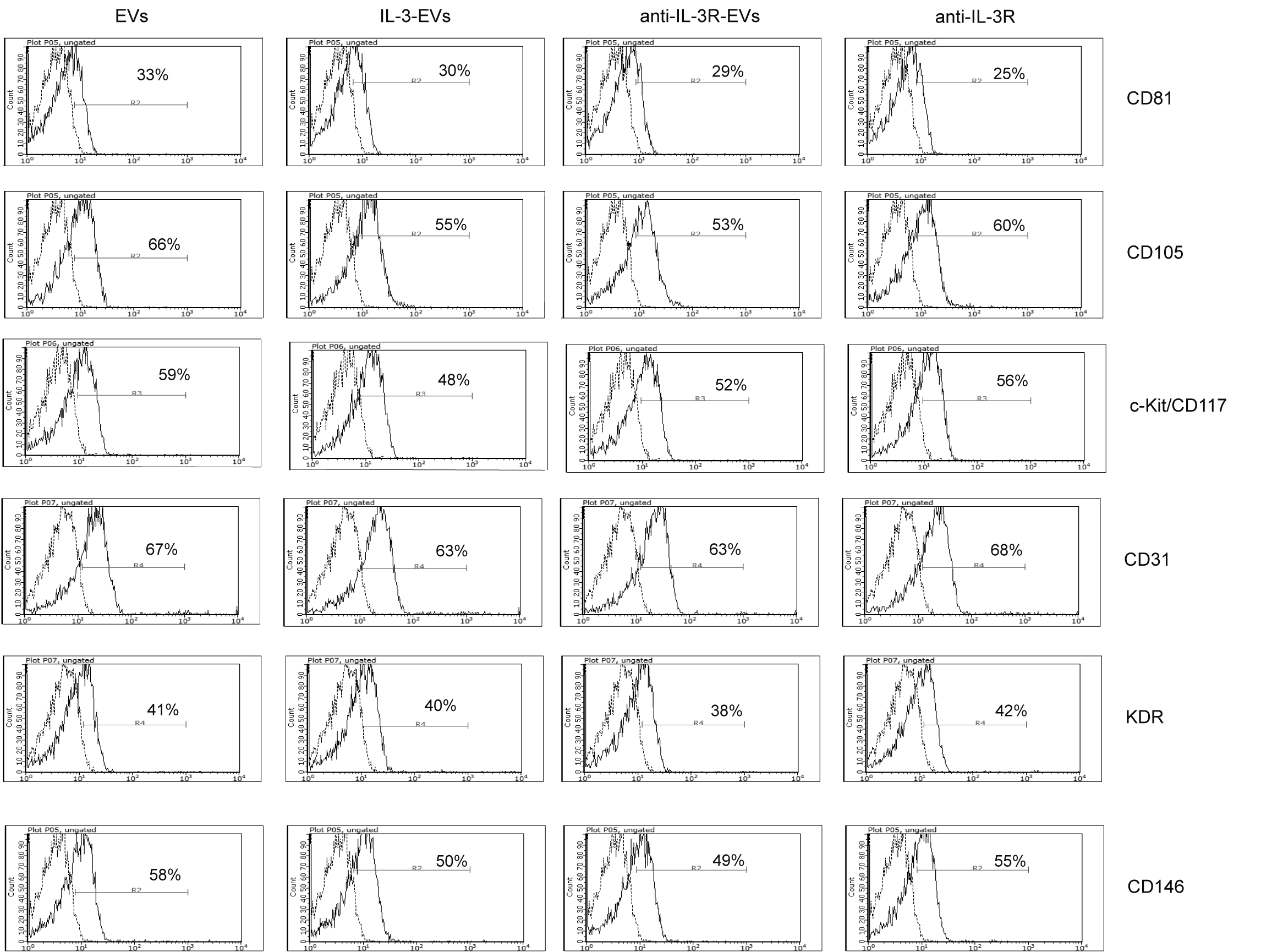
**

**Supplementary Figure S2.** Representative FACS analysis of different EC-derived EVs reported in the histograms as the percentage of CD81, CD105, c-Kit/CD117, CD31, KDR and CD146 expression. Isotype controls were included.

**
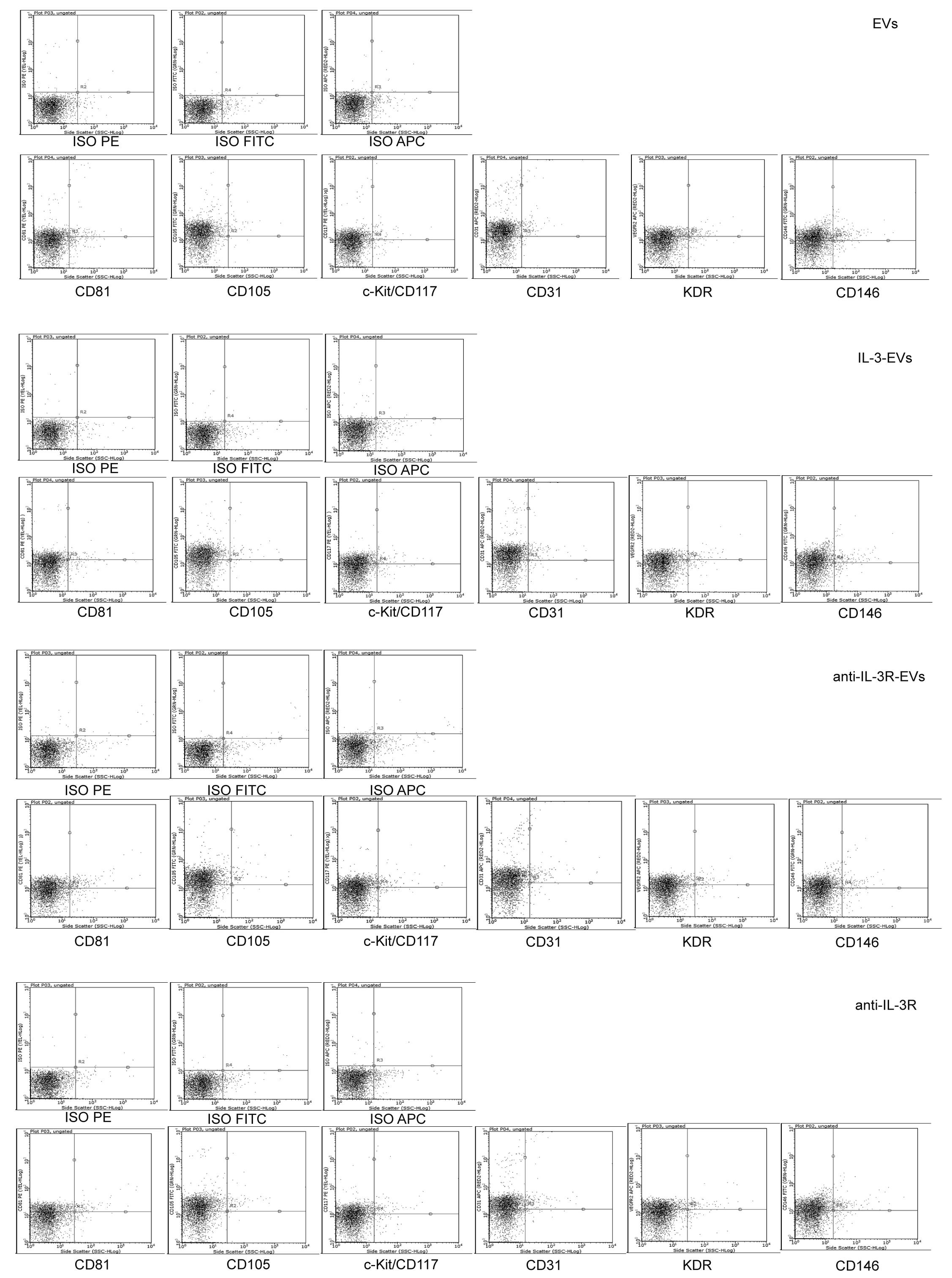
**

**Supplementary Figure S3.** Representative FACS analysis of different EC-derived EVs reported as dot plot analysis for CD81, CD105, c-Kit/CD117, CD31, KDR and CD146. Isotype controls were included.

**
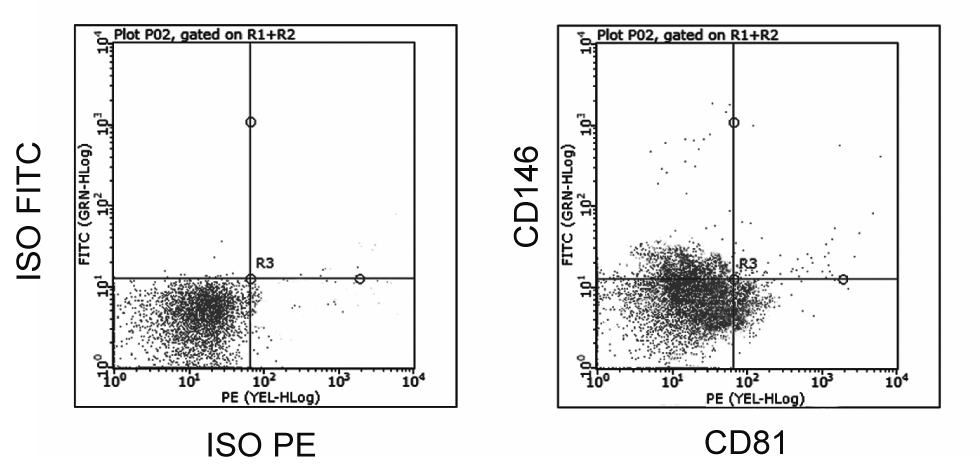
**

**Supplementary Figure S4.** Representative FACS analysis of double immunostaining for CD81 and CD146 markers on IL-3-EVs reported as dot plot analysis. Isotype controls were included. The results are representative of four different experiments (n=4) performed in triplicate (percentage of positivity: CD81=27±3; CD146=46±5; CD81/CD146=0.12±0.1)

**
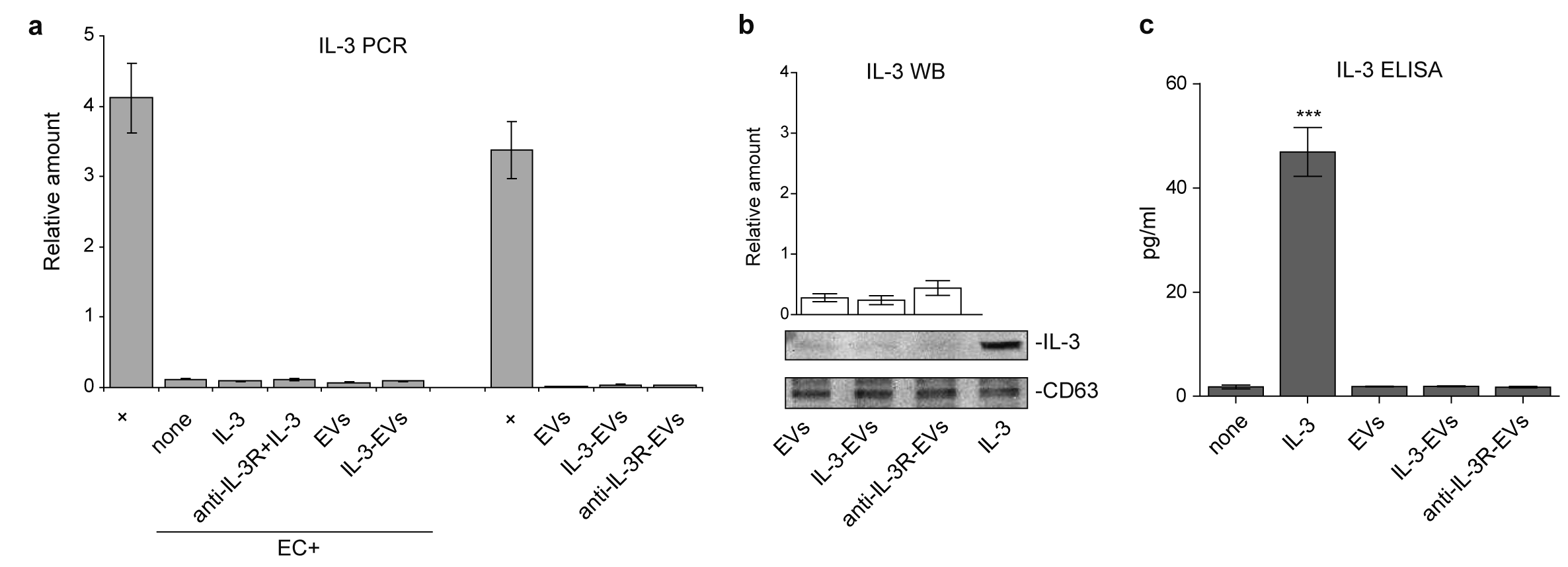
**

**Supplementary Figure S5.** **Absence of IL-3 in IL-3-EVs. (a)** PCR was performed on reversed mRNA extracted from the indicated EC-derived EVs (right panel a) or from IL-3- or EV-treated ECs (left panel a) to evaluate IL-3 mRNA. Total RNA from the MLA cell line of a gibbon was used as positive control for IL-3 amplification primers. **(b)** Protein extracts from the indicated EVs were subjected to SDS-PAGE to evaluate IL-3 content. 2ng of the human recombinant IL-3 was used as positive control. **(c)** An Elisa assay was performed to evaluate membrane bound IL-3 on EVs. All the results are representative of four different experiments (n=4) performed in triplicate.


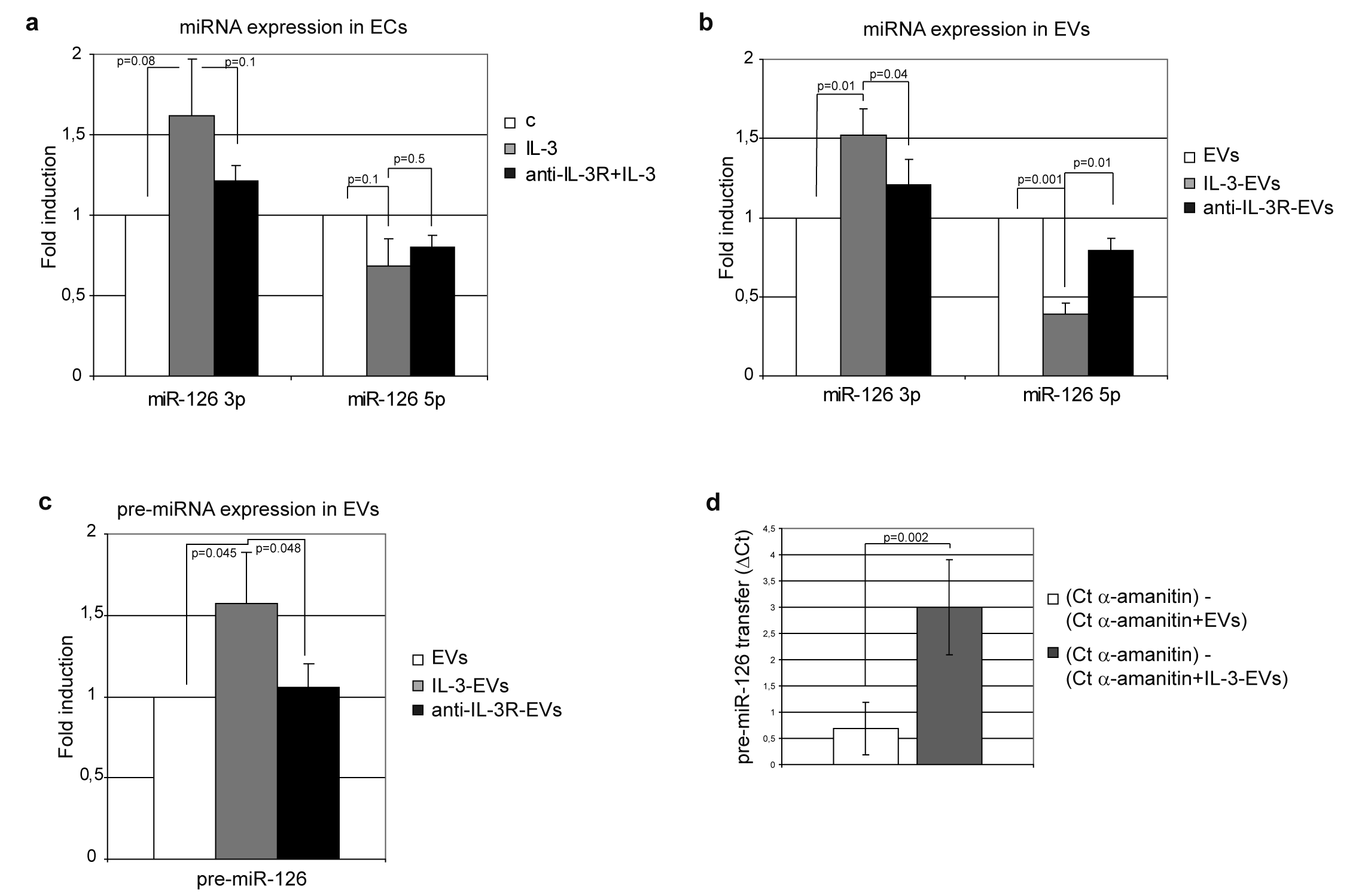


**Supplementary Figure S6**. **miR-126-5p and pre-miR-126 content in EC-derived EVs**. **(a)** miR‐126-3p and miR-126-5p expression was evaluated by quantitative real-time PCR (qRT-PCR) on ECs, untreated (c) or treated with IL-3 (IL-3) in the presence or in the absence of the anti-IL3Ralpha blocking antibody (anti-IL-3R). Data normalized to RNU6B are representative of five different experiments performed in triplicate (n=5) **(b)** miR‐126-3p and miR-126-5p expression was evaluated as above on EVs recovered from ECs, treated as above (*n*=5) (*p=0.01* IL-3-EVs vs EVs; *p=0.04* IL-3-EVs vs anti-IL-3R-EVs for miR-126-3p: *p=0.001* IL-3-EVs vs EVs; *p=0.01* IL-3-EVs vs anti-IL-3R-EVs for miR-126-5p). **(c)** pre-miR‐126 expression was evaluated by qRT-PCR on EVs recovered from ECs, untreated or treated as above (n=5) (*p<0.05* IL-3-EVs vs EVs and anti-IL-3R-EVs). **(d)** ECs incubated in the presence of 50 μg/ml of α-amanitin to inhibit EC transcription were stimulated or not with EVs or IL-3-EVs. EV-pre-miR-126 transfer was evaluated by q-RT-PCR. The difference in Ct values (ΔCt) between α-amanitin-treated ECs alone or with the indicated EVs is reported (*p=0.002*). The results are representative of four different experiments (n=4) performed in triplicate (mean±SD).


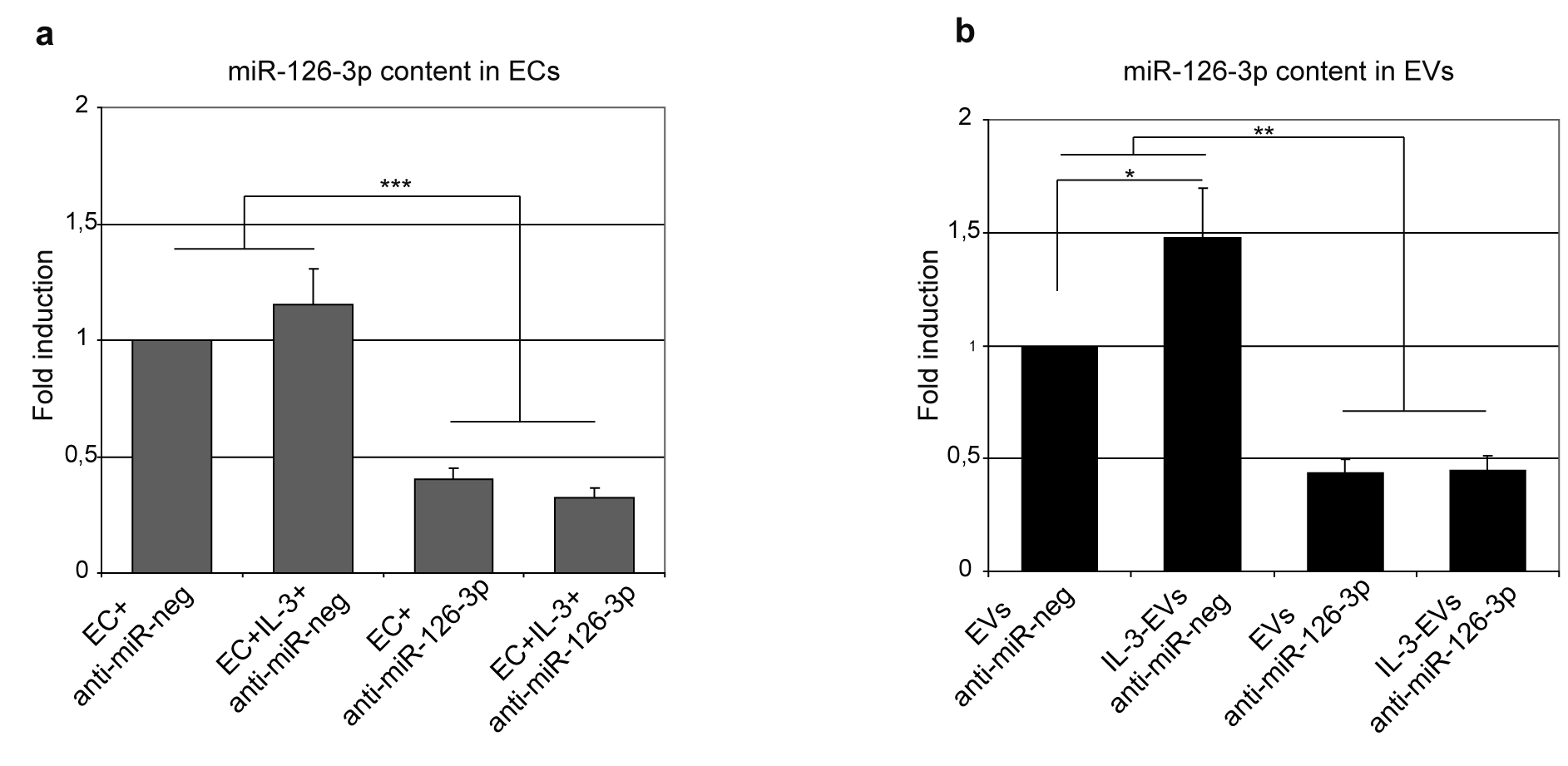


**Supplementary Figure S7. Loss of function experiments for miR-126-3p expression. (a-b)** miR‐126-3p expression was evaluated by qRT‐PCR on ECs that had either undergone transfection with anti-miR-126-3p oligonucleotides or not (a). EVs were isolated from these cells and analyzed by qRT-PCR for their miR-126-3p content (b). Data, normalized to RNU6B, are representative of four different experiments (n=4), performed in triplicate. (****p<0.001* anti-miR-126-3p transfected ECs, alone or plus IL-3, vs anti-miR neg in a; **p<0.05* IL-3-EVs anti-miR- neg vs EVs anti-miR- neg, ***p<0.01* EVs and IL-3-EVs anti-miR-126-3p vs EVs and IL-3-EVs anti-miR neg in b)

**
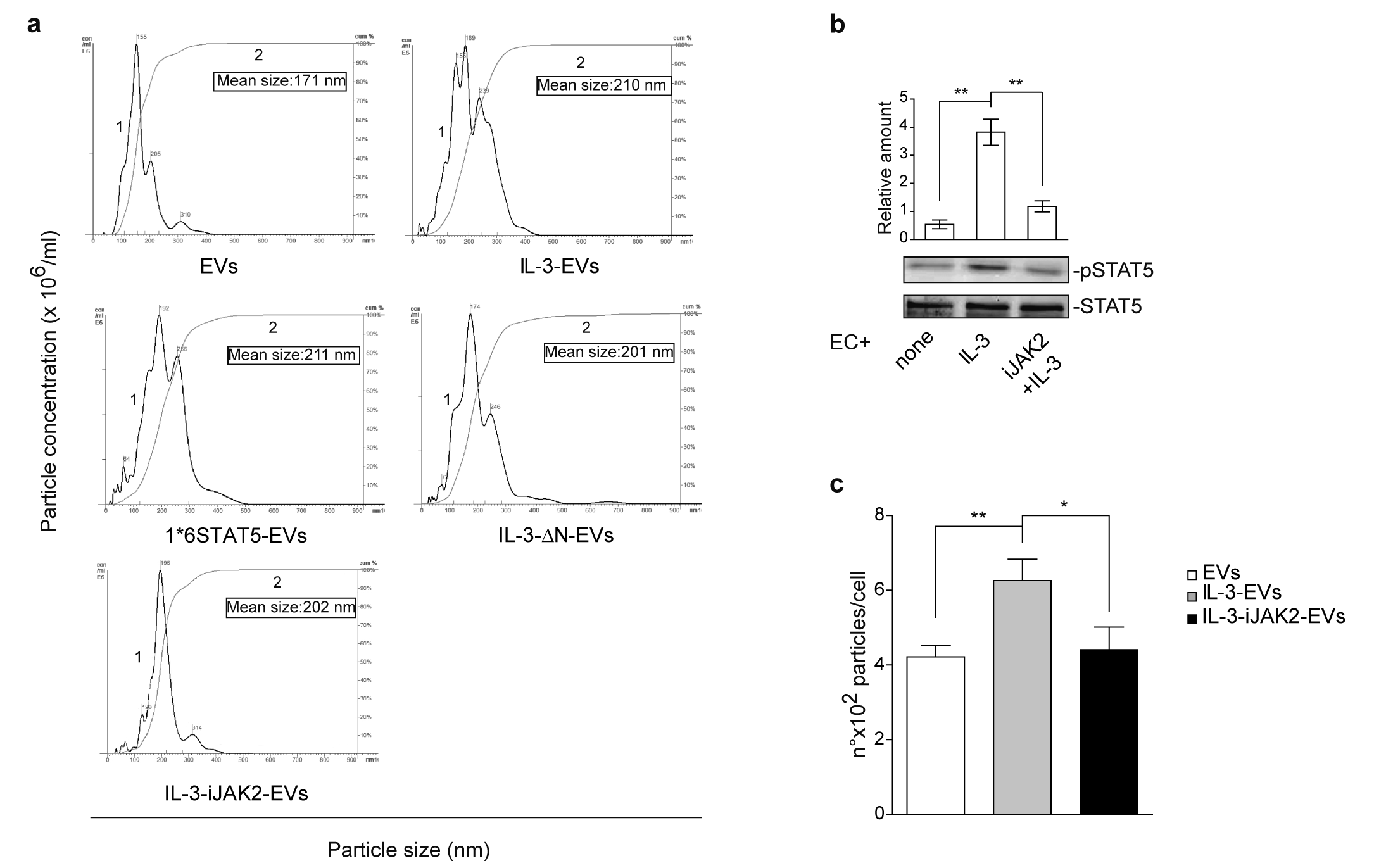
**

**Supplementary Figure S8. (a)** Representative images of NanoSight analyses performed on the 100k fraction of EVs recovered from ECs untreated, IL-3-treated or transfected with ΔNSTAT5 or 1*6STAT5 constructs. The JAK2 inhibitor was also used in ECs treated with IL-3. Curve 1, relationship between particle distribution (left *y* axis) and particle size (*x* axis); curve 2, correlation between cumulative percentage distribution of particles (percentile in right *y* axis) and particle size (*x* axis). **(b)** ECs treated with IL-3 alone or IL-3 plus the anti-JAK2 inhibitor were analyzed for pSTAT5 content and normalized to STAT5 (n=4); (***p<0.01* none and JAK2 inhibitor + IL-3 vs IL-3). **(c)** Number of EV particles (mean±SEM) calculated per cell at isolation. Data refer to EVs from ECs (EVs), IL-3-treated ECs (IL-3-EVs) or from IL-3-treated ECs plus JAK2 inhibitor (IL-3-iJAK2-EVs). The results are representative of four different experiments performed in triplicate (*n*=4) (**p<0.05*, IL-3-iJAK2-EVs vs IL-3-EVs; ***p<0.01* EVs vs IL-3-EVs).
